# A rater agreement study on measurements in cross-sectional CBCT images exploring the association between alveolar bone morphology and craniofacial height

**DOI:** 10.1007/s11282-020-00493-4

**Published:** 2020-12-03

**Authors:** Anna Klinge, Ayman Al-Okshi, Jonas Becktor, Christina Lindh

**Affiliations:** 1 Faculty of Odontology, Department of Oral and Maxillofacial Surgery and Oral Medicine, Malmö University, Malmö, Sweden; 2 Faculty of Dentistry, Department of Oral Medicine and Radiology, Sebha University, Sebha, Libya; 3 Faculty of Odontology, Oral-and Maxillofacial Radiology, Malmö University, 205 06 Malmö, Sweden

**Keywords:** Facial bones, Cephalometry, Radiography, cone beam CT, Observer variation

## Abstract

**Objectives:**

To investigate rater agreement regarding measurements of height and width of the maxilla and mandible using cross-sectional images from CBCT examinations. Furthermore, to explore the association between vertical craniofacial height and alveolar bone morphology.

**Methods:**

Pre-treatment CBCT scans from 450 patients referred for treatment to a private clinic for orthodontics and oral surgery in Scandinavia were available and of these, 180 were selected. Lateral head images were generated from the CBCT volumes to categorise subjects into three groups based on their craniofacial height. Cross-sectional images of the maxillary and mandibular bodies at three locations in the maxilla and mandible, respectively, were obtained and measured at one height and two width recordings by five raters. One-way analysis of variance with a Tukey post hoc test was performed. A significance level of 5% was used.

**Results:**

Rater agreement was mostly excellent or good when measuring height and width of the maxilla and mandible in cross-sectional CBCT images. For height (of the alveolar bone/bodies), there were statistically significant differences between the low- and the high-angle groups for all the observers when measuring in the premolar and midline regions, both in the maxilla and in the mandible.

**Conclusion:**

The high agreement found ensures a reliable measurement technique and confirms the relation between craniofacial height and alveolar bone height and width.

**Supplementary Information:**

The online version contains supplementary material available at 10.1007/s11282-020-00493-4.

## Introduction

Knowledge of the morphological features of the alveolar bone is of great importance for orthodontic tooth movement as well as for the planning and thus, the outcome of dental implant treatment. In orthodontics, movements of teeth in a narrow alveolar bone may cause bone dehiscence, root resorption and gingival recessions, especially in the lower midline region [1]. In implant treatment, sufficient bone volume is a prerequisite for satisfactory outcome and the underlying bone structure plays a key role in the establishment of an acceptable aesthetic result, especially in the anterior maxilla. The bone surrounding an implant site must be of sufficient height and thickness to obtain and keep a harmonious gingival margin [2]. For these clinical situations, assessments and often measurements in radiographs are performed. Before assessment tools can be used for a clinical situation, the reliability of the tool must be established [3] where reliability can be seen as the ability of a measurement to differentiate among subjects or objects. Furthermore, agreement between measurements is an important concept to provide information about the quality of measurements [4].

Several studies have provided evidence that there is a significant association between craniofacial height and the morphology of the alveolar bone [5–7]. A majority of these studies have used lateral cephalometric radiographs to analyse the vertical and the sagittal dimensions of the face [8, 9] and to some extent, these studies have used radiographs obtained with a posterior–anterior projection [9, 10]. The drawbacks of these two imaging techniques are that the three-dimensional structure of an object is imaged two dimensionally which causes a loss of information. The use of tomographic imaging provides the possibility to assess the alveolar bone morphology in three dimensions as well as different regions in detail. Computed tomography (CT) of dry skulls has been used to study the association between mandibular structures and craniofacial type as well as between craniofacial type and bucco-lingual molar inclination [11, 12]. Gracco and co-workers used cone beam CT (CBCT) images of patients to investigate associations between the morphology of the upper jaw, the position of the upper incisors, and craniofacial type as well as the association between the morphology of the mandibular symphysis and the various craniofacial types [13, 14]. Also, based on measurements in CBCT images, significant relationships have been found between craniofacial height and alveolar bone height and width in different tooth-bearing regions of the maxilla and mandible [15, 16]. With regard to measurements of alveolar bone height and width and the association to craniofacial height, some studies present intrarater agreement [11, 15]. However, presentation of interrater agreement is infrequent.

The aim of this study was, therefore, to investigate rater agreement regarding measurements of height and width of the maxilla and mandible using cross-sectional images from CBCT examinations. Furthermore, to explore the association between vertical facial height and alveolar bone morphology.

## Materials and methods

This is a retrospective rater-based study on agreement of measurements of the maxilla and mandible in CBCT images obtained in patients before orthodontic treatment. It was conducted, analysed, and reported in accordance with the Guidelines for Reporting Reliability and Agreement Studies (GRRAS) [4]. An initial study protocol was prepared, including data collection, raters and statistical analyses. The protocol was discussed and accepted by the raters.

### Subjects

Pre-treatment CBCT scans from 450 patients, females over 15 years and males over 16 years, referred for treatment during 2008–2013 to a private clinic for orthodontics and oral surgery in Scandinavia were available. Either CBCT scans from individuals with missing permanent teeth, other than third molars, periodontal disease visually detected on the radiographs, major asymmetries of the jaws or previous orthodontic treatment was excluded.

### Radiography and categorization of subjects

CBCT examinations were performed using an i-CAT CBCT 17–19 (Imaging Sciences International, LLC 1910 N Penn Road, Hatfield, PA 19440, US). The patients were seated in an upright position during scanning. With the aid of laser markers, the midsagittal and occlusal planes were adjusted perpendicular to each other. Field of view (FOV) was set to 16 cm × 13 cm with a voxel size of 0.3 mm. Exposure was set at 120 kVp and 18.54 mAs with a scanning time of 17.8 s. Calibration of this machine was regularly performed according to the manufacturer’s requirements twice a year.

Lateral head images were generated from the CBCT scans using the i-CAT software program. Cephalometric analysis of lateral images was done using the computer software program Total Interactive Orthodontic Planning System [17] (TIOPS, www.tiops.com). Mouse-click on the points of landmarks was used to classify subjects into three groups based on their craniofacial height using the angle of the lower mandibular border (Mandibular line, ML) in relation to cranial base (Nasion-Sella line, NSL). The inclination of the angle formed between the NSL line and the ML line was used to categorize the subjects into the following: low-angle < 27°, average/normal-angle 27–37° and a high-angle group > 37°. After identifying 60 individuals in the low-angle group, this number of scans was set as the limit for the number to be included in the normal- and high-angle group for equal comparisons giving a total of 180 subjects, as described previously (16).

Using i-CAT Vision software (Imaging Sciences International, Hatfield, Pennsylvania, USA), a fully reconstructed three-dimensional image with sagittal, coronal, and axial slices was generated.

### Raters and rating (measurements)

Five raters performed measurements on the CBCT images. Of the raters, one is a specialist in oral and maxillofacial radiology (with 29 years of experience), and one is a post doc in oral and maxillofacial radiology (with 5 years of experience). Furthermore, the raters consisted of one specialist in oral and maxillofacial surgery (with 16 years of experience), one resident at the same department and one general dental practitioner. All raters were aware of the purpose of the study and performed the same measurements independently of each other. Prior to the measurements, an information session and calibration exercise took place with all the raters, and the assessment instructions were specified both verbally and in writing. Thus, the instructions were provided to all the raters. All raters were familiar with handling CBCT images.

All measurement sessions took place in the same room and a BARCO (MFGD 1318; BARCO, Kortrijk, Belgium) 18.10 greyscale liquid crystal display monitor was used with a luminance of 400 cd/m^2^ and resolution of 1280 × 1024 pixels. The observation room was dimly lit and kept constant below 50 lx as recommended by American Association of Physicists in Medicine Task Group 18 [18]. The distance to the screen was approximately 50 cm. There was no restriction on the observation time. The raters were allowed to use the zooming tool. All raters were blinded to clinical features such as craniofacial height and sex.

**Fig. 1:**
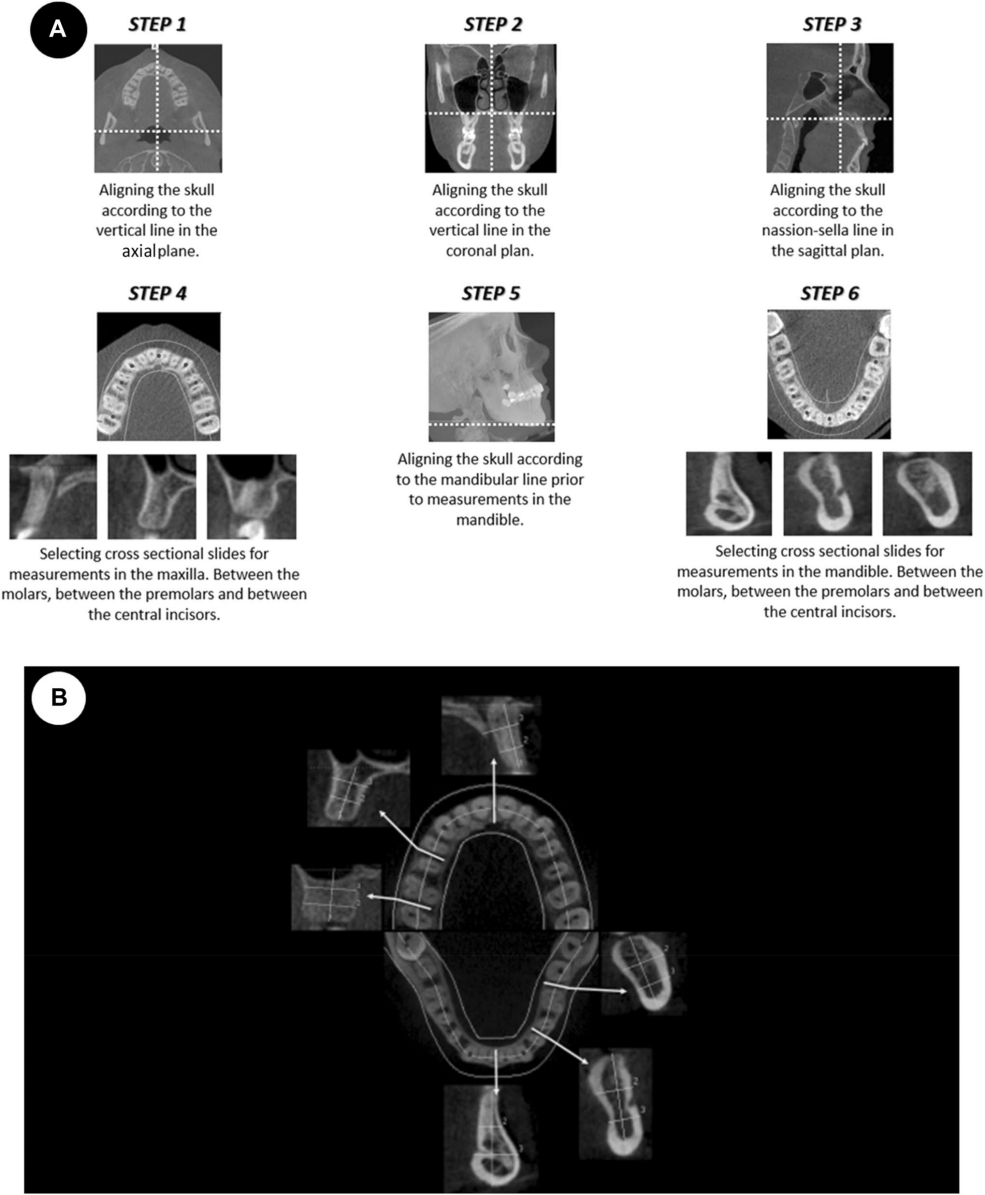
**a** When selecting sites for measurements, the steps performed by the raters when adjusting the volumes for small deviations in the patient´s head position during exposure. **b** Measurements of height and width in cross sectional CBCT images of the maxilla and mandible. One height and two width measurements at each site were measured. The sites were named according to location of the neighboring teeth, e.g. upper molar = UM

Before beginning to measure, raters had the possibility to adjust for small deviations in the patient´s head position during exposure by re-aligning the skull through an adjustment of the images in the sagittal, coronal and axial planes, respectively. The nasion line of the subject was oriented horizontally prior to measurements in the maxilla. For mandibular measurements, the mandibular base line was set horizontally. For every group of patients (low, normal, high angle) 3 sites (molar, premolar, and midline region) in maxilla and mandible, respectively, were measured by each rater in rotation (Fig. 1a). Sites were chosen within the three groups of patients to obtain an even distribution between molar, premolar and midline regions. Measurements were performed, with one height and two width measurements between the teeth at selected cross-sectional sites (Fig. 1b). The measurements were performed using the measurement tools in the software program i-CAT vision. For calculation of intrarater agreement, 10% of the sites were randomly selected in IBM SPSS software (version 22.0; IBM Corp Armonk, NY, USA) and measured by all raters in a second session after approximately 2 months.

The measurements were simultaneously and manually documented in an Excel (Microsoft Office Excel^®^ 2010; Microsoft Corporation, Redmond, WA) file by the responsible researcher.

### Data analysis

All computations necessary for the statistical analysis were performed using IBM SPSS software (Version 22.0; IBM Corp Armonk, NY, USA). For all variables, the three groups (low, normal, high angle) were compared using a one-way analysis of variance with a Tukey post hoc test. A significance level of 5% was used in all comparisons.

Inter- as well as intrarater agreement of measurements in selected cross-sectional sites was calculated as intra-class correlation coefficients (ICCs 2.1) with 95% confidence interval (CI). Only measurements from the first measurement session performed by each rater were used to calculate interrater ICC. The level of agreement was interpreted according to the guideline proposed by Koo and Li [3] as follows: < 0.50, poor; between 0.50 and 0.75, fair; between 0.75 and 0.90 good; above 0.90, excellent agreement.

## Results

Characteristics of individuals belonging to the three groups of craniofacial height is seen in Table 1. Height and width of the maxillary and mandibular alveolar bone/bodies were measured by all raters at all selected sites giving a total of 1080 measurements per rater. For calculation of intrarater agreement, re-measurements were performed by all raters at 10% of the sites giving 108 measurements per rater.

**Table 1:** Characteristics of individuals from which CBCT scans were obtained belonging to one of three groups of craniofacial height (*n* = number)

	Craniofacial height		
	Low angle *n*, mean age years (range)	Normal angle *n*, mean age years (range)	High angle *n*, mean age years (range)
Female *n* = 117	38, 30.1 (15–70)	44, 25.5 (15–58)	35, 26.2 (15–49)
Male *n* = 63	22, 28.8 (17–47)	16, 32.0 (16–71)	25, 23.7 (16–43)

### Interrater agreement

**Fig. 2:**
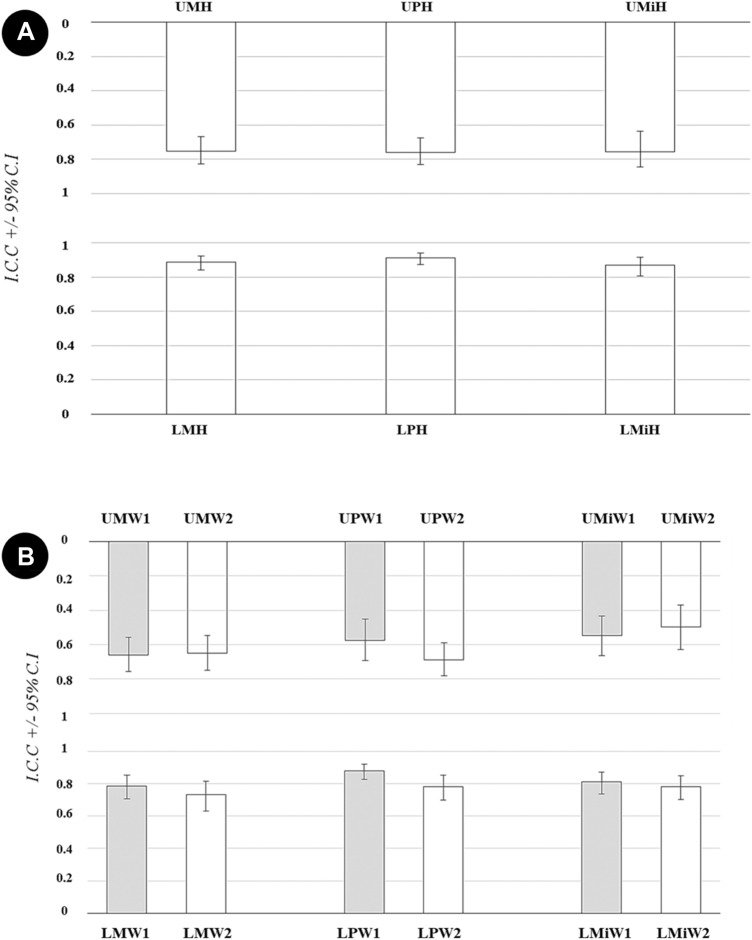
**a** Overall interrater agreement expressed as Intraclass Correlation Coefficient (ICC) and Confidence Intervals (CI) for measurements of the height of maxillary and mandibular body in cross sectional CBCT images. **b** Overall interrater agreement expressed as ICC and CI for measurements of the width of maxillary and mandibular body in cross sectional CBCT images. *UMH* upper molar height, *UPH* upper premolar height, *UMiH* upper midline height, *LMH* lower molar height, *LPH* lower premolar height, *LMiH* lower midline height, *W1* coronal width, *W2* apical width, *UM* upper molar, *UP* upper premolar, *UMi* upper midline, *LM* lower molar, *LP* lower premolar, *LMi* lower midline

Overall interrater agreement ICC for height measurements was in general excellent or good and varied between 0.75 and 0.91 (CI 0.67–0.83 and 0.88–0.94) depending on the measured site. The values were higher for measurements in the mandible compared with the maxilla, and the highest value was recorded in the mandibular premolar region (Fig. 2a).

Corresponding ICC values for width measurements were in general lower than for height measurements. In all sites but one, coronal measurements showed higher ICC than apical measurements. Overall interrater agreement ICC for coronal measurements varied between 0.55 and 0.88 (CI 0.43–0.66 and 0.83–0.92) with the highest value in the mandibular premolar region. For apical measurements, ICC varied between 0.50 and 0.78 (CI 0.37–0.63 and 0.70–0.85) with the highest value also being found in the mandibular premolar region (Fig. 2b).

For pairwise interrater, the highest ICC values were also achieved for measurements in the mandible compared with the maxilla. The values for height measurements were in general higher and when comparing coronal and apical width measurements, the highest values were seen for coronal measurements. Taking all pairwise interrater agreements into consideration, 8% was interpreted as excellent, 47% as good, 39% as fair and 5% as poor agreement according to the suggested guidelines for interpretation of ICC values by Koo and Le [3] (Supplementary Table S1).

### Intrarater agreement

**Fig. 3:**
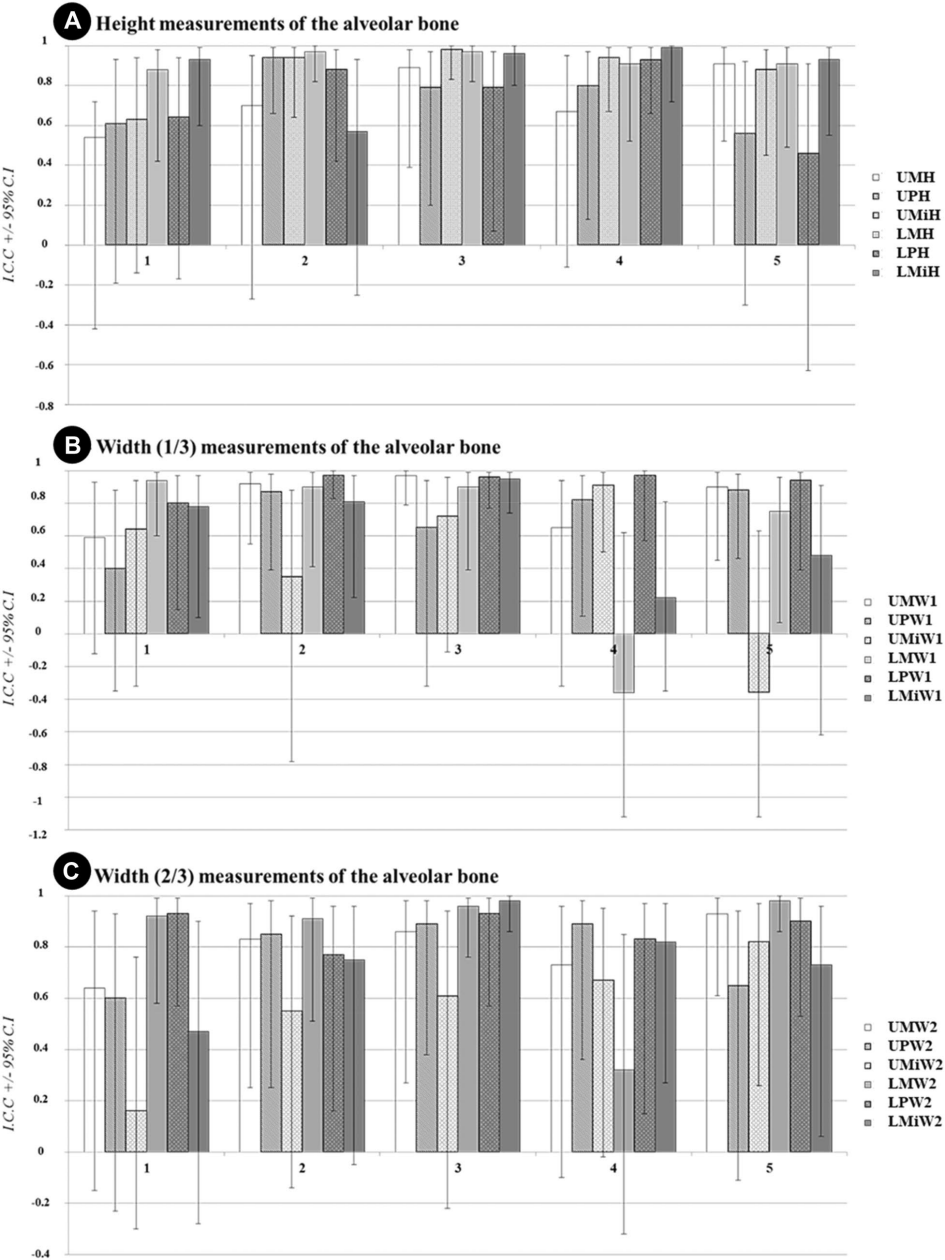
Intrarater agreement of five raters (1–5) expressed as Intraclass Correlation Coefficient (ICC) and Confidence Intervals (CI) for the measurements of (**a**) cross-sectional height measurement; (**b**) coronal width (1/3) measurement and (**c**) apical width (2/3) measurement of maxillary and mandibular body in cross-sectional CBCT images. *W1* coronal width, *W2* apical width, *UM* upper molar, *UP* upper premolar, *UMi* upper midline, *LM* lower molar, *LP* lower premolar, *LMi* lower midline

No rater consistently presented the highest or the lowest intrarater agreement, but the CI was somewhat wider for some raters. The highest agreement was found in the molar region in the mandible when measuring the height of the alveolar bone (Fig. 3).

### Height and width measurements related to craniofacial height

#### Height measurements

Statistically significant differences between the low- and high-angle groups for all raters were found for measurements in the premolar and midline regions, both in the maxilla and in the mandible. Regarding the molar region in the maxilla, there were significant differences between the low- and high-angle groups for three of the raters. Regarding the height measurement in the molar region in the mandible, there were no significant differences between any of the groups for any of the five raters (Table 2).

**Table 2:** Height Measurements in CBCT cross-sectional images of the maxilla and mandible of individuals with different craniofacial height performed by five raters

Site	Rater	Craniofacial height					
		High-angle group	Normal-angle group	Low-angle group	Significance		
		Mean in mm (SD)	Mean in mm (SD)	Mean in mm (SD)	High-normal	Normal-low	High-low
Maxilla							
Molar	1	16.6 (2.8)	15.6 (2.0)	15.7 (2.6)	0.428	0.991	0.505
	2	16.3 (2.8)	15.4 (2.7)	14.8 (2.8)	0.338	0.797	0.107
	3	17.5 (2.8)	16.2 (2.4)	14.9 (2.5)	0.258	0.265	0.007*
	4	16.8 (2.7)	15.2 (2.5)	14.0 (2.5)	0.140	0.332	0.004*
	5	16.9 (2.5)	15.6 (2.6)	14.7 (2.6)	0.272	0.499	0.025*
Premolar	1	20.0 (3.0)	18.4 (1.9)	16.6 (3.1)	0.165	0.109	0.001*
	2	19.8 (2.8)	18.3 (1.9)	16.9 (3.0)	0.195	0.188	0.002*
	3	20.2 (3.4)	18.6 (1.6)	16.4 (2.4)	0.100	0.025*	0.000*
	4	20.0 (2.6)	18.4 (2.1)	16.6 (2.4)	0.102	0.056	0.000*
	5	20.0 (2.7)	18.2 (2.4)	16.3 (2.1)	0.000*	0.000*	0.000*
Midline	1	22.4 (3.0)	21.0 (2.6)	22.4 (2.6)	0.257	0.220	0.005*
	2	20.5 (2.8)	19.8 (2.4)	17.4 (2.7)	0.701	0.016*	0.002*
	3	21.1 (2.6)	20.3 (2.3)	18.0 (2.4)	0.533	0.011*	0.000*
	4	20.1 (3.2)	19.2 (2.3)	19.2 (2.3)	0.493	0.209	0.016*
	5	22.9 (2.7)	20.6 (2.3)	18.3 (2.7)	0.000*	0.000*	0.000*
Mandible							
Molar	1	27.4 (3.9)	26.7 (2.3)	26.0 (2.3)	0.737	0.729	0.296
	2	27.3 (4.3)	26.5 (2.3)	25.7 (2.5)	0.754	0.654	0.255
	3	26.9 (4.4)	26.7(2.4)	25.6 (2.4)	0.966	0.544	0.396
	4	27.0 (3.8)	26.3 (2.4)	25.6 (2.5)	0.746	0.711	0.289
	5	26.7 (3.3)	26.6 (2.8)	26.2 (2.6)	0.993	0.683	0.611
Premolar	1	32.3 (2.7)	31.3 (2.7)	28.0 (2.8)	0.515	0.001*	0.000*
	2	32.8 (2.7)	31.5 (2.6)	28.7 (3.1)	0.282	0.010*	0.000*
	3	32.6 (3.0)	31.3 (2.9)	28.3 (3.4)	0.362	0.011*	0.000*
	4	33.1 (2.6)	31.9 (2.8)	28.5 (3.1)	0.387	0.001*	0.000*
	5	32.1 (3.6)	30.7 (3.0)	28.7 (3.4)	0.025*	0.001*	0.000*
Midline	1	32.7 (3.3)	31.6 (3.5)	29.8 (2.4)	0.489	0.168	0.012*
	2	33.4 (2.7)	33.3 (4.1)	30.9 (2.8)	0.993	0.064	0.049*
	3	33.6 (2.6)	32.3 (3.2)	30.4 (3.0)	0.330	0.119	0.003*
	4	32.7 (2.9)	31.4 (2.9)	30.0(2.2)	0.309	0.187	0.005*
	5	35.3 (3.3)	32.3 (3.0)	29.5 (2.8)	0.000*	0.000*	0.000*

Mean values, standard deviation (SD) and significant differences between groups

*p < 0.05

#### Width measurements

*Coronal width* measurements in the *maxilla* at molar, premolar and midline regions displayed no statistically significant differences between any of the groups for the five raters. When measuring in the *mandible*, there were no statistically significant differences between the three facial groups when measuring in the molar region. When measuring in the premolar region, statistical differences were seen between the low- and high-angle groups for one rater. But when measuring in the midline region, there were statistically significant differences between the low- and high-angle groups for four out of five raters (Table 3).

**Table 3:** Coronal width measurements in CBCT cross-sectional images of the maxilla and mandible of individuals with different craniofacial height performed by five raters

Site	Rater	Craniofacial height					
		High-angle group	Normal-angle group	Low-angle group	Significance		
		Mean in mm (SD)	Mean in mm (SD)	Mean in mm (SD)	High-normal	Normal-low	High-low
Maxilla							
Molar	1	14.7 (1.9)	14.0 (1.0)	14.0 (1.9)	0.387	1.0	0.391
	2	14.5 (1.5)	14.3 (0.8)	13.8 (1.6)	0.904	0.461	0.244
	3	15.0 (1.8)	14.6 (0.9)	14.4 (1.2)	0.684	0.858	0.368
	4	14.9 (1.8)	14.1 (1.0)	14.2 (1.5)	0.150	0.972	0.228
	5	14.2 (1.6)	14.1 (1.3)	13.7 (1.5)	0.953	0.425	0.272
Premolar	1	9.2 (1.4)	9.5 (1.1)	9.1 (1.0)	0.664	0.516	0.969
	2	9.6 (1.3)	9.5 (1.1)	9.3 (1.3)	0.974	0.808	0.680
	3	10.2 (1.6)	10.0 (1.3)	10.2 (1.8)	0.967	0.937	0.995
	4	9.7 (1.5)	9.8 (1.2)	9.8 (1.4)	0.954	0.989	0.900
	5	9.0 (1.3)	9.3 (1.1)	9.2 (1.2)	0.208	0.655	0.690
Midline	1	6.5 (1.2)	6.6 (1.3)	6.9 (1.2)	0.954	0.747	0.567
	2	7.2 (1.5)	6.7 (0.8)	6.9 (1.4)	0.386	0.853	0.711
	3	7.2 (1.6)	6.8 (1.0)	7.2 (1.0)	0.534	0.592	0.995
	4	6.7 (1.1)	6.9 (1.1)	7.3 (1.2)	0.785	0.451	0.155
	5	6.9 (1.0)	7.1 (1.0)	6.9 (1.4)	0.652	0.721	0.993
Mandible							
Molar	1	13.2 (1.3)	12.9 (1.8)	13.3 (1.8)	0.756	0.731	0.999
	2	13.2 (1.4)	13.3 (2.0)	13.6 (1.4)	1.0	0.763	0.745
	3	13.7 (1.4)	13.5 (2.0)	13.0 (2.1)	0.967	0.675	0.522
	4	13.0 (1.8)	13.1 (2.4)	13.5 (1.9)	0.966	0.825	0.680
	5	13.0 (1.9)	13.3 (1.9)	13.3 (2.0)	0.677	0.970	0.529
Premolar	1	9.4 (1.9)	10.9 (1.6)	10.5 (2.2)	0.055	0.822	0.613
	2	9.4 (1.7)	10.9 (1.5)	10.6 (2.2)	0.038*	0.873	0.116
	3	9.9 (2.1)	11.2 (1.7)	11.5 (2.4)	0.154	0.870	0.053
	4	9.6 (1.9)	10.9 (1.7)	11.1 (2.4)	0.102	0.944	0.500
	5	9.5 (1.6)	10.4 (2.0)	10.5 (2.0)	0.025	0.914	0.008*
Midline	1	6.9 (1.8)	8.2 (2.1)	8.6 (1.6)	0.083	0.820	0.020*
	2	7.1 (2.3)	7.7 (1.9)	8.1 (1.4)	0.622	0.800	0.267
	3	6.9 (1.6)	8.2 (1.9)	8.5 (1.3)	0.031*	0.888	0.009*
	4	6.5 (1.6)	8.2 (2.0)	8.3 (1.3)	0.006*	0.956	0.003*
	5	6.4 (1.5)	7.3 (1.6)	8.2 (1.6)	0.004*	0.006*	0.000*

Mean values, standard deviation (SD) and significant differences between groups

*p < 0.05

*Apical width* measurement displayed no statistically significant differences between any of the three craniofacial groups when measuring in the molar, premolar, and midline region in the maxilla. No statistical differences were presented between any of the craniofacial groups in the molar and premolar region in the mandible. Although, in the incisal mandibular region, there were statistically significant differences between the low and high facial groups for the majority of the raters, four out of five (Table 4).

**Table 4:** Apical width measurements in CBCT cross sectional images of the maxilla and mandible of individuals with different craniofacial height performed by five raters

Site	Rater	Craniofacial height					
		High-angle group	Normal-angle group	Low-angle group	Significance		
		Mean in mm (SD)	Mean in mm (SD)	Mean in mm (SD)	High-normal	Normal-low	High-low
Maxilla							
Molar	1	16.0 (2.6)	14.9 (1.2)	16.8 (3.5)	0.405	0.058	0.550
	2	15.7 (2.6)	15.5 (1.2)	15.1 (2.1)	0.941	0.786	0.583
	3	16.2 (2.5)	16.1 (1.7)	15.6 (2.8)	0.987	0.984	1.0
	4	16.0 (2.4)	15.4 (1.3)	15.6 (2.5)	0.656	0.971	0.793
	5	16.1 (2.4)	15.9 (1.9)	15.9 (2.1)	0.913	1.0	0.919
Premolar	1	9.92 (1.7)	10.2 (1.6)	10.6 (1.8)	0.893	0.737	0.458
	2	10.5 (1.9)	10.4 (1.6)	10.7 (2.2)	0.995	0.927	0.960
	3	10.7 (1.9)	10.8 (1.6)	11.4 (2.3)	1.0	0.590	0.577
	4	10.7 (2.0)	10.8 (1.8)	11.3 (2.3)	0.970	0.779	0.637
	5	10.4 (2.2)	10.4 (1.9)	10.6 (2.0)	0.976	0.908	0.804
Midline	1	13.3 (4.1)	12.6 (2.6)	12.1 (3.2)	0.744	0.886	0.456
	2	10.8 (3.2)	11.7 (3.2)	10.4 (3.0)	0.598	0.347	0.903
	3	12.8 (2.3)	12.8 (2.4)	12.5 (3.1)	0.998	0.910	0.933
	4	13.0 (3.1)	13.5 (2.09	13.4 (2.8)	0.799	0.998	0.833
	5	12.6 (2.1)	12.0 (1.9)	12.3 (2.5)	0.231	0.724	0.657
Mandible							
Molar	1	11.0 (1.5)	11.3 (1.3)	11.4 (1.7)	0.793	0.989	0.711
	2	10.8 (1.4)	11.0 (1.2)	11.1 (1.8)	0.959	0.956	0.842
	3	11.4 (1.5)	11.6 (1.2	12.3 (2.1)	0.960	0.276	0.172
	4	11.0 (1.3)	11.3 (1.4)	11.2 (1.6)	0.837	0.980	0.925
	5	10.6 (1.4)	11.0 (1.79	10.9 (1.6)	0.420	0.888	0.706
Premolar	1	9.0 (1.6)	10.1 (1.9)	9.4 (1.5)	0.086	0.343	0.731
	2	9.4 (2.0)	10.1 (1.6)	9.4 (1.6)	0.343	0.406	0.992
	3	10.2 (1.8)	10.4 (1.7	10.1 (1.8)	0.904	0.849	0.993
	4	9.4 (2.2)	10.1 (1.8)	10.0 (1.6)	0.423	0.983	0.527
	5	9.4 (1.8)	10.2(1.7)	10.0 (1.7)	0.045	0.833	0.164
Midline	1	12.1 (2.3)	13.7 (2.5)	14.4 (2.0)	0.072	0.589	0.006*
	2	11.9 (2.3)	13.1 (2.59	14.1 (1.7)	0.208	0.359	0.008*
	3	12.2 (2.2)	13.8 (2.6)	14.7 (1.9)	0.075	0.404	0.002*
	4	12.0 (2.4)	13.4 (2.5)	13.5 (1.8)	0.131	0.996	0.110
	5	12.3 (2.1)	13.5 (2.4)	14.1 (1.9)	0.005*	0.282	0.000*

Mean values, Standard Deviation (SD) and significant differences between groups

*p < 0.05

## Discussion

The present study demonstrates that it is possible to achieve good agreement between several raters as well as within raters when height and width of the alveolar bones are measured in cross-sectional CBCT images. Furthermore, an association between craniofacial height and alveolar bone height found in this study was statistically significant for all five raters’ measurements.

Accuracy is one part of investigating the strength of a diagnostic method, the other is the agreement, which is the degree to which scores, or ratings, are identical [4]. Unfortunately, agreement studies are generally neglected and do not appear in the different stages of evaluating studies of diagnostic methods or in studies where diagnostic methods are used to evaluate treatment outcomes [19, 20]. The results of a study of an imaging method and clinical problem will be influenced not only by the number of objects and raters but also by the rater selection, e.g. their expertise [21]. The raters in the present study represented professional experience from different fields of expertise and the length of their experience varied; this is also the case with all potential users in a clinical situation. Since the raters may have different other prior experience and visual concepts, a study with several raters can be anticipated to give a more reliable result. To avoid influence on the assessments and consequently the result, the raters were blinded to all patient information and to the other raters’ measurements.

The raters received instructions to choose an approximal cross-sectional site between first and second molar, between first and second premolar and in the midline at which to perform the height and width measurements. This may have given a lower ICC score and wider confidence intervals than if pre-selected sites had been used. On the other hand, this situation mimics the clinical situation better as “free selection” takes place in a clinical situation. It is important to be able to apply the results in a clinical setting as the external validity would be limited if the results were only applicable in a staged research environment.

Although a standardised calibration procedure to prevent bias was applied prior to measurements, a variation in rater agreement was found. Overall interrater agreement was in general higher for measurements in cross-sectional CBCT images of the mandible compared with the maxilla and it was the highest in the premolar region for both height and width measurements. This indicates that anatomical landmarks in the mandible might be easier to identify. The marginal bone area and basis of the mandible are probably more distinct in an image than the marginal bone area in the maxilla and the borders to maxillary sinus and nasal cavity. Pairwise interrater agreement was the highest for measurements at mandibular sites and even if agreement varied a majority of the agreements were in the category “good agreement” (Supplementary Table S1) [3]. The lowest agreement was noted when the raters measured in the upper midline region. This might be explained by difficulties in the interpretation of sites where there are anatomical variations such as the incisive foramen.

Interrater agreement was expressed as overall ICC as well as pairwise interrater agreement. The pairwise interrater calculations is able to detect if any rater differs considerably from the others, which can then be analysed further. The difference might be due to a misinterpretation of the instructions or an unfavourable measurement technique. In this study, no clear deviation was observed for any rater (Supplementary Table S1).

The CI of the ICC was in some measurements negative which indicates considerable uncertainty. The somewhat low rater agreement can be explained by variations in the steps performed by the raters when adjusting for small deviations in the patient’s head positioning and in the selection of sites for measurements as well as difficulties in identifying anatomical structures and handling CBCT volumes. In the study by Sadek et al., inter- and intrarater agreement was reported as ICC with paired t test [15]. However, no confidence interval was reported, and the number of observers was vaguely described as “other orthodontists” without mentioning their exact numbers or professional experience. Regardless of the statistical approach used, confidence intervals as measures of statistical uncertainty should be reported to allow the readers to be able to determine, in particular, the lower level of reliability/agreement.

Taking several rater measurements into consideration, the results of this study showed that patients with large craniofacial height (high angle) has a significantly higher alveolar bone, both in the maxilla and in the mandible compared to those with low craniofacial height (low angle). The association between craniofacial height and cross-sectional maxillary and mandibular bone height was most evident in the premolar and incisal regions. These results strengthen the findings of our previous study [16] and are to a certain extent in concordance with the results of the study by Sadek et al. [15] where statistical differences were found in the anterior part of the maxilla. Therefore, it can be concluded that the dentoalveolar compensatory mechanism, via continued tooth eruption, responds in the maxilla and mandible by enlarging the vertical size of the frontal dentoalveolar heights in long-face subjects and, conversely, less tooth eruption will take place in short-face subjects [22].

A further indication, on the association between vertical craniofacial height and alveolar bone morphology, especially in the anterior region, is that in this study the coronal and apical width in the midline region of the mandible (LO-MID) was narrower in the group with high craniofacial height compared with the group with low craniofacial height. In the premolar and molar areas, there were no statistically significant differences in the coronal nor in the apical width measurements between any of the groups.

### Strength and limitations

Rater agreement has been investigated to explore measurement errors and variations in interpretation, which affects the value of measurements in clinical practice [23] and have to be taken into account when evaluating methods in any diagnostic yield. The number of subjects in the study is larger than that included in other comparable studies [15, 24]. Nevertheless, it is a retrospective study design where CBCT examinations were performed prior to the design of the current study. The subjects included in the study were referred to a specialist clinic for orthodontic and oral surgery which means that the results may not be generally applicable.

## Conclusions and clinical implications

Knowledge of the morphological features of the alveolar bone is of importance when planning orthodontic tooth movement or dental implant treatment. The results from this study show the significant association between craniofacial height and alveolar bone dimensions as explored by several raters and would provide reference data that can be useful prior to orthodontic or dental implant treatment in subjects with different craniofacial types.

## Supplementary Information

Below is the link to the electronic supplementary material.

Supplementary file1 (DOCX 33 KB)
