# Supplementary material for: A rater agreement study on measurements in cross-sectional CBCT images exploring the association between alveolar bone morphology and craniofacial height
Source: Oral Radiol. 2020 Dec 3;37(4):573–84. doi: 10.1007/s11282-020-00493-4 (PMC8448720; doi:10.1007/s11282-020-00493-4)
Supplement: Supplementary file 1 — Supplementary file1 (DOCX 33 KB) [file 11282_2020_493_MOESM1_ESM.docx]

**SUPPLEMENTARY TABLE S1** Pairwise interrater agreement expressed as Intraclass Correlation Coefficient (ICC) with 95% confidence interval (CI)

| *Height measurements* | | | | | | | | | | | | | | | | | | | | | | | | |
| --- | --- | --- | --- | --- | --- | --- | --- | --- | --- | --- | --- | --- | --- | --- | --- | --- | --- | --- | --- | --- | --- | --- | --- | --- |
|  | 1 | | | | | | 2 | | | | | | 3 | | | | | | 4 | | | | | |
|  | *UM* | *UP* | *UMi* | *LM* | *LP* | *LMi* | *UM* | *UP* | *UMi* | *LM* | *LP* | *LMi* | *UM* | *UP* | *UMi* | *LM* | *LP* | *LMi* | *UM* | *UP* | *UMi* | *LM* | *LP* | *LMi* |
|  | | | | | | | | | | | | | | | | | | | | | | | | |
| 2 | 0.61  (0.42-0.74) | 0.83 (0.72-0.89) | 0.67  (0.19-0.85) | 0.87  (0.79-0.92) | 0.93  (0.87-0.96) | 0.82 (0.56-0.91) |  |  |  |  |  |  |  |  |  |  |  |  |  |  |  |  |  |  |
| 3 | 0.67  (0.50-0.79) | 0.69  (0.52-0.80) | 0.75 (0.44-0.88) | 0.81 (0.71-0.88) | 0.99 (0.82-0.93) | 0.91 (0.78-0.96) | 0.86  (0.76-0.92) | 0.80  (0.68-0.87) | 0.85  (0.74-0.91) | 0.90  (0.83-0.94) | 0.89  (0.82-0.93) | 0.84 (0.74-0.90) |  |  |  |  |  |  |  |  |  |  |  |  |
| 4 | 0.61 (0.42-0.75) | 0.82 (0.72-0.90) | 0.60 (0.090-0.81) | 0.83 (0.73-0.90) | 0.90 (0.80-0.94) | 0.93 (0.88-0.95) | 0.82  (0.72-0.90) | 0.83  (0.72-0.89) | 0.79 (0.66-0.87) | 0.91 (0.86-0.95) | 0.90  (0.84-0.94) | 0.77  (0.53-0.88) | 0.84  (0.63-0.92) | 0.71 (0.56-0.82) | 0.79  (0.62-0.88) | 0.86  (0.77-0.91) | 0.88  (0.80-0.93) | 0.89 (0.76-0.94) |  |  |  |  |  |  |
| 5 | 0.54 (0.33-0.70) | 0.69 (0.54-0.81) | 0.76 (0.60-0.86) | 0.88 (0.81-0.93) | 0.94 (0.90-0.97) | 0.88  (0.72-0.94) | 0.82  (0.72-0.89) | 0.81 (0.70-0.88) | 0.82 (0.61-0.91) | 0.97 (0.95-0.98) | 0.97 (0.95-0.80) | 0.86 (0.78-0.92) | 0.88 (0.79-0.93) | 0.71  (0.56-0.82) | 0.88 (0.80-0.93) | 0.90  (0.85-0.94) | 0.92  (0.86-0.95) | 0.92  (0.88-0.95) | 0.83  (0.73-0.90) | 0.71 (0.56-0.82) | 0.73 (0.45-0.86) | 0.93  (0.88-0.96) | 0.92  (0.87-0.95) | 0.89  (0.70-0.95 |
| *Coronal width measurements* | | | | | | | | | | | | | | | | | | | | | | | | |
|  | | | | | | | | | | | | | | | | | | | | | | | | |
| 2 | 0.54  (0.34-0.70) | 0.68 (0.51-0.79) | 0.53  (0.32-0.69) | 0.87  (0.79-0.92) | 0.94  (0.90-0.96) | 0.77  (0.64-0.85) |  |  |  |  |  |  |  |  |  |  |  |  |  |  |  |  |  |  |
| 3 | 0.62 (0.42-0.76) | 0.37  (0.11-0.59) | 0.46  (0.24-0.64) | 0.71  (0.56-0.82) | 0.85  (0.65-0.93) | 0.81  (0.70-0.88) | 0.70  (0.48-0.83) | 0.42  (0.18-0.61) | 0.62  (0.44-0.75) | 0.74  (0.60-0.84) | 0.90 (0.71-0.96) | 0.67 (0.50-0.79) |  |  |  |  |  |  |  |  |  |  |  |  |
| 4 | 0.65 (0.48-0.78) | 0.64  (0.42-0.78) | 0.50 (0.29-0.67) | 0.74 (0.61-0.84) | 0.83  (0.73-0.90) | 0.87 (0.79-0.92) | 0.60 (0.41-0.74) | 0.77  (0.64-0.86) | 0.55 (0.34-0.71) | 0.81  (0.70-0.88) | 0.85 (0.76-0.91) | 0.77  (0.65-0.86) | 0.76 (0.83-0.85) | 0.49 (0.28-0.66) | 0.52  (0.31-0.69) | 0.69 (0.53-0.80) | 0.88 (0.80-0.93) | 0.84 (0.75-0.95) |  |  |  |  |  |  |
| 5 | 0.64 (0.46-0.77) | 0.65 (0.48-0.78) | 0.57 (0.37-0.72) | 0.84 (0.72-0.90) | 0.92 (0.87-0.95) | 0.89 (0.56-0.96) | 0.66 (0.49-0.78) | 0.81 (0.69-0.88) | 0.52 (0.31-0.68) | 0.89 (0.82-0.93) | 0.94 (0.90-0.96) | 0.82 (0.70-0.89) | 0.74 (0.32-0.88) | 0.40 (0.12-0.61) | 0.62 (0.43-0.75) | 0.81 (0.70-0.88) | 0.86 (0.69-0.93) | 0.78 (0.58-0.88) | 0.72  (0.55-0.83) | 0.74 (0.50-0.86) | 0.59 (O.40-0.73) | 0.80  (0.68-0.87) | 0.84 (0.74-0.90) | 0.87 (0.77-0.93) |
| *Apical width measurements* | | | | | | | | | | | | | | | | | | | | | | | | |
|  | | | | | | | | | | | | | | | | | | | | | | | | |
| 2 | 0.52 (0.30-0.68) | 0.78 (0.65-0.86) | 0.48 (0.20-0.70) | 0.78  (0.65-8.7) | 0.84 (0.74-0.90) | 0.76 (0.63-0.85) |  |  |  |  |  |  |  |  |  |  |  |  |  |  |  |  |  |  |
| 3 | 0.59 (0.40-0.73) | 0.63  (0.38-0.78) | 0.47 (0.25-0.65) | 0.55 (0.34-0.71) | 0.63 (0.36-0.79) | 0.83 (0.74-0.90) | 0.72  (0.50-0.84) | 0.59 (0.40-0.73) | 0.59 (0.17-0.79) | 0.64 (0.26-0.82) | 0.74 (0.52-0.85) | 0.81 (0.67-0.88) |  |  |  |  |  |  |  |  |  |  |  |  |
| 4 | 0.56 (0.36-0.71) | 0.69 (0.45-0.82) | 0.44 (0.22-0.63) | 0.81 (0.70-0.88) | 0.77 (0.63-0.86) | 0.67 (0.51-0.79) | 0.76 (0.62-0.85) | 0.76 (0.62-0.85) | 0.40 (0.02-0.65) | 0.84 (0.74-0.90) | 0.88 (0.81-0.93) | 0.70 (0.54-0.81) | 0.72 (0.56-0.83) | 0.59 (0.40-0.73) | 0.48 (0.26-0.65) | 0.59 (0.34-0-75) | 0.72 (0.57-0.83) | 0.71 (0.55-0.82) |  |  |  |  |  |  |
| 5 | 0.55 (0.35-0.71) | 0.77 (0.65-0.86) | 0.54 (0.34-0.70) | 0.78 (0.65-0.86) | 0.76 (0.63-0.85) | 0.88 (0.81-0.93) | 0.71 (0.52-0.83) | 0.83 (0.72-0.89) | 0.55 (0.29-0.73) | 0.89 (0.81-0.93) | 0.90 (0.83-0.94) | 0.86 (0.78-0.92) | 0.82 (0.72-0.89) | 0.56 (0.57-0.71) | 0.58 (0.39-0.73) | 0.70 (0.46-0.83) | 0.73 (0.56-0.84) | 0.84 (0.74-0.90) | 0.65 (0.48-0.78) | 0.73 (0.57-0.83) | 0.50 (0.25-0.68) | 0.84 (0.75-0.90) | 0.86  (0.77-0.91) | 0.75 (0.61-0.84) |

*UM,* upper molar*; UP,* upper premolar*; UMi,* upper midline*; LM,* lower molar*; LP,* lower premolar*; LMi,* lower midline

ICC <0.50=poor agreement (total: 10st **≈** 5%)

ICC 0.50–0.75=fair agreement (total: 71st **≈** 39%),

ICC >0.75–0.90=good agreement (total: 84st **≈** 47%),

ICC >0.90–1.0=excellent agreement, (total: 15st **≈** 8%, of 180st)
